# Doctors and Chemists

**Published:** 1888-10-06

**Authors:** David Walsh


### DOCTORS AND CHEMISTS.

By David Walsh, L.R.C.P. and S., Edin., etc.

A discussion has lately been going on in the columns of a daily paper as to the way in which doctors and chemists encroach upon each other's fields. Such a title is begging the question, for while the doctors have legal right to do chemists' work, yet if the latter do doctors' work they no longer encroach, but are committing a legal trespass, and if life is endangered by that illegal act they commit a felony. Medical men dispense, and are entitled to do so by the law, which has tested their competency; chemists, on the other hand, have no legal right to examine patients and prescribe. The doctors are using a right of way, while the chemists are trespassers and poachers. To go a little more into detail, the position of affairs between these two bodies—which stand somewhat in the relation of a profession and a trade—is the following: Any medical man who cares to do so may dispense for himself, or otherwise he can employ a chemist to make up his prescriptions. The druggist is a mere purveyor or compounder of drugs, and if he goes beyond that to inquire into symptoms, and to say what are the fit and proper remedies to be used, he does so at his peril, and becomes an unqualified practitioner of a most dangerous order, namely, one which possesses a smattering of knowledge. It is as though a bricklayer, a mere handler of bricks and mortar, were to assume the airs and pretensions of an architect. But there is this difference, that whereas the mason deals with dead and inanimate matter, the prescribing chemist is trifling with a thing no less sacred and complex than human life and its machinery. Government recognises the fact that protection against unqualified medical practitioners is necessary for the well-being of the subject. Hence the charters granted to our colleges and universities, by which they become corporate bodies with the power of granting licences to practise medicine to such candidates as show they have the proper amount of special knowledge. Before becoming fully qualified the medical practitioner is obliged to undergo an expensive education and pay many fees. He has, therefore, a right to look to Government for the protection that the mutual transaction certainly seems to imply. The authorities, however, do nothing whatever to punish unqualified practice. Everything has to be done by individual action, or by the Apothecaries' Hall, which appears to be the only medical body that has the legal right to undertake a prosecution. Moreover, it is no easy matter to secure a conviction, even in the face of the clearest evidence. The feeling of both magistrates and juries is often in favour of the prescribing chemist. Mr. Thomas Laffan, of Cashel, who has written much on the subject, says: "The Apothecaries' Act of 1815 contained a clause—the well-known 28th—which has been utilised to score some successes against practising chemists. One of the best-known cases, and the most commented upon, is the well-known one of the 'Apothecaries' Hall v. Wiggins.' He was charged with examining a patient, and by doing so violating the clause in question, which reserved such business for the medical practitioners, among whom the apothecary was by the Act itself then for the first time included. The 28th Section reserved to druggists the right to do whatever they had been in the habit of doing prior to '15. It appeared from the evidence that the chemists' business, prior to that Act, was confined to preparing and selling drugs, and the adventuresome chemist was beaten. In other cases, however, similar actions were defeated, the juries taking a totally different view of the custom before '15, and basing their verdicts as much upon the notion that slight cases might be treated by druggists, as upon evidence produced before them—that correspondingly trifling examinations were in the habit of being made before the date of the Act."

Public opinion has always been more or less on the side of the chemists, and this particular form of unqualified practice seems to have its attractions for every class of society. Many educated people will go to a chemist for a bottle of physic to treat some trifling ailment, and would think themselves very hardly used if they were not allowed to do so. Cheapness, no doubt—that powerful lever of modern times—has a good deal to answer for in this matter. A man with a slight cold will prefer to get a bottle of cough mixture from the nearest chemist to consulting a medical man at heavier fee, with the chance of running up a bill for further attendance; but be this as it may, without going to extremes one may hold that it is the duty of Government to protect the public against themselves, and in the particular instance under discussion to take such measures that shall make the chemist pause before he ventures outside his work of vending or of making up drugs according to directions. The compounder may be an intelligent man, but that fact renders him the more dangerous, because he is likely to be more meddlesome. The argument that a druggist must know something of the art of medicine because he has spent all his days in a drug-shop, reminds one of another personage whose claims are supported by the same weak-kneed process of reasoning. The individual spoken of is the "handy" woman, who undertakes confinements because, having had a large family herself, she "knows all about it," an assurance which seems to be quite satisfactory to herself and her clients, and together they brave dangers before which a professor of the art might quail. Every medical man in general practice must be familiar with women of this kind, and the mischief they work; how alcohol is the mainstay for themselves, the mother, and often for the child; how the infant is swathed up, and pinched about, and physicked, and fed with gruel and arrowroot and butter and other abominations; and how many lives are either ruined or sacrificed by their gross ignorance. The unqualified midwife endangers life by trespassing into fields that Government has long ago set apart for a certain specially-educated class, whose knowledge in an art that requires much skill has been tested by a corporation. The chemist who lays down his vials to investigate his customer's disease is trespassing on another branch of the rights of the same corporate body, and both druggist and "handy" woman are alike endangering the safety of the subject. The symptoms of many diseases are obscure at their commencement, and it is often of the utmost importance that treatment should be skilful and immediate. To detect the first stages of such affections is not infrequently a very difficult matter, requiring long practice and training, and it is absurd, on the face of it, to imagine that a mere compounder of drugs should stand behind his counter and pick up diagnosis. That art can be acquired only after long years of close study and systematic training of the powers of reason, memory, and observation. No candid man will deny that in many cases the chemist does well enough, but that fact does not alter the main argument. A drug vendor has no right to undertake treatment on his own responsibility, and although he sometimes do as well as anyone else, yet he is always liable to grave mistakes through his want of a special technical knowledge that he cannot pretend to have acquired.

A certain druggist, who freely confesses to prescribing, and who defends his conduct stoutly on various grounds, has done many things quite openly in the presence of the writer, and as there was never any question of confidence, some few of his doings may be laid before the admiring gaze of the readers of this article. A carman walked into the shop one day with a damaged forefinger, from which about a square inch of skin had been scraped away in its whole thickness. Under any circumstances it was an awkward wound, which at the best would take some time to heal, but the actual treatment was as follows. There was a smart oozing of blood, which the assistant tried to staunch by cold and pressure. Not succeeding in the attempt, however, he handed the case over to his master. That worthy, after looking at the injury with the eagle glance common to the half-educated, called for some strong liquor ferri perchloridi, and poured a liberal allowance of that powerful caustic over the raw surface. To say that the man suffered martyrdom would be committing oneself to a mild statement of fact. An antiseptic and absorbent dressing would have set the finger right in a few weeks, whereas by this heroic measure much of the neighbouring tissue would be inevitably destroyed, and a deep wound caused instead of the original superficial one. Poor fellow! he paid sixpence for that piece of surgery, but it must have cost him a good deal more in the end than the half-crown fee of a qualified surgeon. Moreover, there was a public hospital within a stone's throw, where his finger would have been dressed for nothing at all. Another day, as the writer entered the shop, a well-dressed man limped slowly and painfully away, and the ever-cheerful proprietor volunteered the information that his patient was suffering from an attack of acute gout, showing me his prescription with no little pride. It was a Gatling gun, indeed, quite calculated to finish off a weakly patient on the spot. "I don't give cheap drugs like soda and gentian," said the honest fellow with a glow of pride. "I use drugs with something in them. I want my patients to come back to me again." To enable his gouty customer to achieve that desirable object, his physic contained about six depresants, among which the writer recalls colchicium, aconite, iodide, and bromide of potassium, all very good things in their way, but when given together in large doses, sufficient to tame and subdue a veritable Samson.

But it is surely time to cry out for strong measures when non-professional people meddle with so delicate an organ as the eye, where the damage done is often immediate and beyond recall. Every surgeon connected with ophthalmic hospitals will be able to say that a certain proportion of cases are sure to crop up in which the sight has been lost through treatment of chemists. They have one remedy for all eye affections—an astringent—and to use that in many conditions means almost certain destruction of the sight. The tissues that make up the eye are so delicate and change so rapidly, that early skilled treatment means more than half the battle. It is true that in the event of injury to his customer the chemist lays himself open to an action for damages; but prosecutions do not take place, people continue to bring their ruined sight to the infirmaries, and the game still goes on. One patient the writer has pressed to bring an action, but he refused on the ground that the chemist "had done his best and he was not going to turn against him," a forgiveness that brings us to what after all lies at the root of the matter. In reforming this abuse, as in most others, public opinion must be first educated. Let the people be fully informed as to the danger of going for medical advice to those who are not qualified to give it. Meanwhile, it is the duty of medical men, of coroners, and of the press, both lay and medical, to point out the risks that are thereby incurred; to bring home cases, to urge on inquests, and to punish offenders. Can the outside public be aware of the fact that a large number of their fellow-countrymen are every year disabled by the loss of their eyesight, solely and simply owing to the unskilful and illegal treatment of chemists? As things stand, ignorance and prejudice are the bulwarks from behind which chemists physic her Majesty's lieges, and laugh in the face of the colleges.

### NEW PREPARATIONS.

Cocoa.—Some time ago we gave an account of cocoa in its crude state, of its uses as food and as a remedial agent, and of several well-known preparations then and now in the market. The competition of the times brings steady pressure upon producers in the direction of cheapness in price and improvement in quality. Messrs. Rowntree, of York, an old-established and well-known Yorkshire firm, have recently put a new cocoa on the market, which they denominate the "Elect Extract." The title presumably means that they have tried every known method used for the preparation of extracts, and that they have proved one particular process the best. That process they use in the manufacture of their new cocoa. Supposing them to have hit upon the best-known method of extraction, and that they use only the best crude materials, there is good ground for concluding that they have produced, and now offer for sale, an article of the first quality. A sample has been submitted to us, which we have tested in the ordinary way. The cocoa is undoubtedly strong, and of a fine flavour. It is soluble in an unusual degree, and ought speedily to take rank among the best preparations of its class.

